# Comparing the long non-coding RNA expression profiles of skeletal muscle and kidney tissues from patients with diabetes

**DOI:** 10.1371/journal.pone.0274794

**Published:** 2022-09-26

**Authors:** Young-Kook Kim

**Affiliations:** Department of Biochemistry, Chonnam National University Medical School, Hwasun, Jeollanam-do, Republic of Korea; Nippon Medical School, JAPAN

## Abstract

**Background:**

Diabetes causes the dysregulation of several organs, and these effects are often closely associated with changes in the expression of long non-coding RNAs (lncRNAs), a group of non-coding RNAs, within these tissues. Previous studies have described a variety of changes in the expression profile of several lncRNAs from different organs in response to the pathogenesis of diabetes. However, none of these studies compared the expression profiles of these lncRNAs between these organs. This study was designed to identify common and specific lncRNAs involved in the progression of diabetes in the skeletal muscles and kidneys.

**Methods:**

Publicly available RNA sequencing data of diabetic patients was obtained from the Gene Expression Omnibus database. By analyzing the expression of lncRNAs in these datasets, differentially expressed lncRNAs in each tissue between non-diabetic and diabetic patients were identified. To identify any lncRNAs changed in common in both kidney and muscle tissues, those lncRNAs that are significantly dysregulated in both datasets were selected.

**Results:**

These evaluations identified a series of novel lncRNAs unique to each organ and several transcripts that were common to both skeletal muscle and kidney tissues in these patients. Interestingly, the genomic location of these lncRNAs suggests that they reside in close proximity to several protein-coding genes known to be related to diabetes suggesting that these lncRNAs may have a regulatory relationship with their neighboring genes.

**Conclusion:**

These results offer valuable insights into the role of lncRNAs during the pathogenesis of diabetes.

## Introduction

Diabetes affects various organs including skeletal muscles, which play an important role in pathogenesis because of their key function in energy homeostasis [1–4]. This is because type 2 diabetics experience an impaired insulin response which reduces oxidative metabolism and limits the amount of fatty acid converted to glucose resulting in increased levels of circulating free fatty acids. These fatty acids then accumulate within the muscle cells, reducing insulin-stimulated glucose uptake. This impairment is usually described as insulin resistance [5]. Because the skeletal muscle is the major tissue responsible for glucose accumulation and disposal, changes in these tissues are known to have an important role in patients with insulin resistance and diabetes [1, 6]. Although research is underway to analyze changes in various tissues including skeletal muscle from a molecular perspective in the context of metabolic diseases [7–11], the detailed molecular mechanism underlying this process still needs to be elucidated.

Long-term diabetes results in the destruction of the small blood vessels and the destruction of the glomerulus vessels in the kidney resulting in reduced kidney function and eventually diabetic nephropathy [12]. Diabetic nephropathy is characterized by an increase in urine albumin and a reduction in kidney function. Many ongoing studies are focused on identifying the factors responsible for these effects in an effort to produce novel therapeutics designed to prevent disease progression. However, to date, there is still no detailed molecular mechanism describing the underlying pathogenesis of diabetic nephropathy.

Diabetes also affects various other organs including the liver, fat, and intestine resulting in their transcriptomic evaluation to understand the underlying mechanisms of diabetic pathogenesis [2–4]. However, despite this, our understanding of the interplay between these organs remains limited, often limiting our ability to fully understand the complexities of this disease.

Long non-coding RNAs (lncRNAs) are a large class of non-coding RNA transcripts of more than 200 nucleotides in length. Many of these lncRNAs reside near protein-coding genes, and a portion of these lncRNAs share overlapping promoter sequences with their neighboring protein-coding genes often being transcribed in the opposite direction, suggesting that these lncRNAs and their neighboring protein-coding genes might be modulated by common transcription factors [13]. In other cases, lncRNAs may share an overlapping sequence with a protein-coding gene producing a duplex RNA structure and sense-antisense pair [13, 14]. The formation of duplex structures could act as a regulatory mechanism for these lncRNAs allowing them to modulate the expression of their protein-coding gene counterpart. Several studies have reported that lncRNAs are likely to play some regulatory role in the pathogenesis of diabetes [15], but the comparison of the role(s) and identities of these lncRNAs in different tissues has not yet been completed. Since diabetes is a disease related to various tissues throughout the body, discovering a lncRNA that functions commonly in multiple tissues in relation to diabetes will provide important clues in the treatment of these diseases.

Given this, this study was designed to compare the expression of specific lncRNAs in myotube and kidney samples from patients with diabetes to identify specific lncRNAs dysregulated in the skeletal muscle and kidney and those that were common to both organs. Subsequent evaluation of the genomic context of these transcripts and their relationship with neighboring protein-coding genes suggests that these transcripts were likely to be connected to diabetic pathogenesis. This study will help to identify specific biomarkers and potential therapeutic targets for the evaluation and treatment of the pathogenic effects of diabetes in different organs.

## Materials and methods

Dataset GSE81965 from the Gene Expression Omnibus (GEO) database [16] includes the RNA sequencing data of myotubes differentiated from skeletal muscle precursor cells, which were collected from patients with different disease conditions: non-obese, obese, patients with type 2 diabetes, and obese patients with type 2 diabetes [17]. These data were then evaluated as previously described [18]. Briefly, the FASTQ sequences were applied to Trimmomatic which removed any sequences of low-quality [19] and the remaining sequences were aligned to the human genome (hg19) using STAR [20]. Then Cuffnorm was used to calculate the Fragments Per Kilobase of transcript per Million mapped reads (FPKM) [21] based on the GENCODE annotation [22]. Those lncRNAs with an average FPKM value of lower than 1 or those with 0 in any sample were removed from further analysis. Then the FPKM values of each of these lncRNAs were compared between the four evaluation groups and those most closely associated with diabetes were identified. Specifically, the lncRNA with significant differences in their expression between non-diabetic and diabetic patients (*P* < 0.01) were selected.

Dataset GSE142025 includes the RNA sequencing data from the kidney biopsies of patients with diabetic nephropathy. This dataset includes three groups of samples collected from patients described as normal, the early phase of diabetic nephropathy, and the advanced phase of diabetic nephropathy [23]. These lncRNAs were then evaluated as described above and lncRNAs were selected for evaluation by comparing the FPKM values for lncRNA expression in the normal and advanced phases of diabetic nephropathy groups. The lncRNAs with significant differences in their expression were selected by using both criteria of the transcripts included in the top 5% based on *P*-value and those with the top 20% based on average expression level.

To select the commonly dysregulated lncRNAs in both skeletal muscle and diabetic nephropathy datasets, the top 5% of significantly changed lncRNAs based on the *P*-value of the expression change were selected from each dataset. Then, those lncRNAs that exist in common in both selected lists were chosen.

## Results and discussion

**Fig 1:**
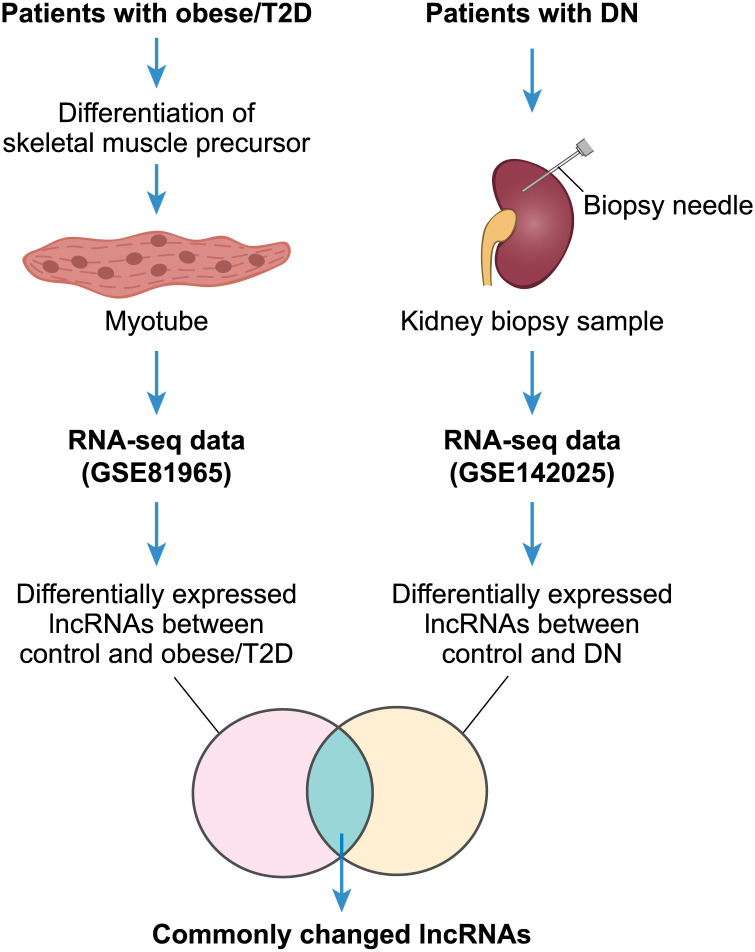
Analysis procedure to measure long non-coding RNAs (lncRNA) expression in the myotubes and kidney tissues of patients with diabetes. See the main text for details.

**Fig 2:**
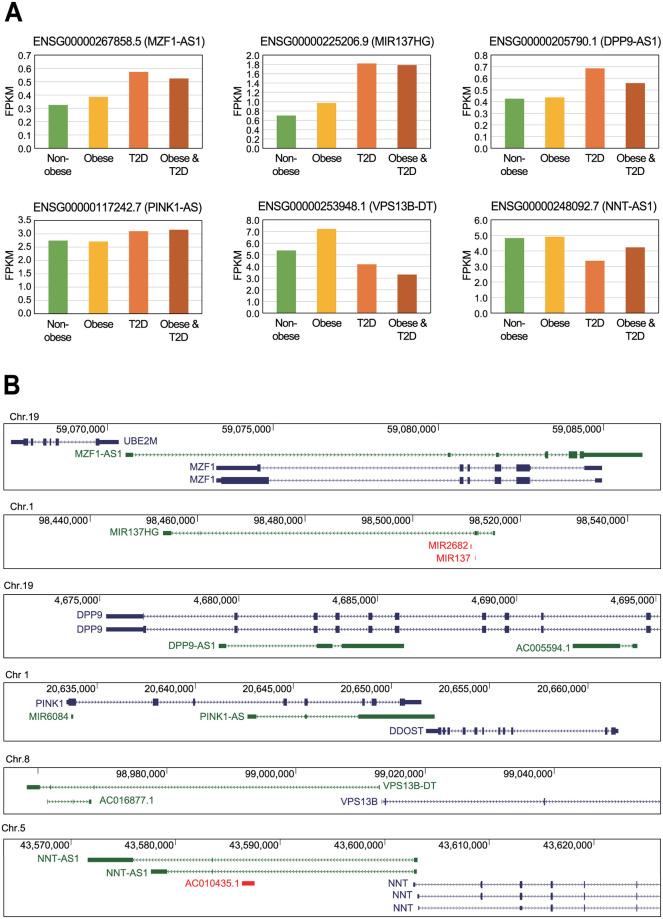
Selected dysregulated long non-coding RNAs (lncRNAs) identified in the myotubes of patients with diabetes. (A) The expression of specific lncRNAs in each of the sample groups was calculated based on the data available in the public GSE81965 dataset, and is reported as Fragments Per Kilobase of transcript per Million mapped reads (FPKM) values. (B) Schematics describing the genomic context of each of the lncRNAs as reported in Genome Browser [24]. Blue lines represent protein-coding genes, green lines lncRNA genes, and red lines small non-coding RNA genes.

A set of publicly available RNA sequencing data for both kidney and muscle tissues of diabetic patients from the GEO database was obtained [16] and used to identify differentially expressed lncRNAs in each condition (Fig 1). The skeletal muscle data was collected from the GSE81965 dataset which evaluated differentiated myotubes from human subjects with type 2 diabetes and/or obesity [17]. After analyzing the expression of each lncRNA in this dataset 19 lncRNAs with significant differences in expression between non-diabetic and diabetic patients (*P* < 0.01) were identified (Table 1). Of these six had been previously described as being associated with diabetes (Fig 2), with four, including MZF1 antisense RNA 1 (MZF1-AS1), MIR137 host gene (MIR137HG), DPP9 antisense RNA 1 (DPP9-AS1), PINK1 antisense RNA (PINK1-AS), demonstrating increased expression in the samples from the patients with type 2 diabetes when compared to the healthy control. The other two previously described lncRNAs, VPS13B divergent transcript (VPS13B-DT) and NNT antisense RNA 1 (NNT-AS1), demonstrated decreased expression in diabetic samples (Fig 2A).

**Table 1:** Expression levels for each of the significantly altered lncRNAs from the skeletal muscle tissues of patients with diabetes.

Gene ID	Non-obese	Obese	T2D	Obese & T2D	Description	Reference (PMID)
ENSG00000236540.7_5	0.9	0.9	1.5	1.4	antisense to *TANGO2*	
ENSG00000228544.1_4	0.4	0.2	0.6	0.5	divergent to promoter of *RABL6*	
ENSG00000267858.5_5	0.3	0.4	0.6	0.5	divergent to promoter of *UBE2M*	26606528,30592156
ENSG00000234793.1_4	1.4	1.3	2.5	2.4	antisense to intron of *D2HGDH*	
ENSG00000273084.1_4	0.7	1.0	1.5	1.4	antisense to promoter of *LOC100129484*	
ENSG00000250988.7_5	6.3	5.4	3.6	3.9	*SNHG21*	
ENSG00000223768.2_3	4.6	4.7	6.0	6.2	divergent to promoter of *POFUT2*	
ENSG00000271976.1_4	2.4	2.4	1.6	1.8	antisense to intron of *IL17RB*	
ENSG00000270604.5_3	0.8	0.9	0.4	0.2	*HCG17* (divergent to promoter of *TRIM39*)	
ENSG00000251257.2_4	14.1	12.1	7.2	5.8	antisense to *EGFLAM*	
ENSG00000264575.1_3	2.2	2.2	1.8	1.6	RNA fragment?	
ENSG00000225206.9_4	0.7	1.0	1.8	1.8	*MIR137HG*	27497953
ENSG00000279080.1_4	0.0	0.0	0.0	0.0	too low expression	
ENSG00000205790.1_4	0.4	0.4	0.7	0.6	*DPP9-AS1* (antisense to DPP9)	16186403,26242871
ENSG00000117242.7_5	2.7	2.7	3.1	3.2	*PINK1-AS1* (antisense to PINK1)	17567565, 33546409
ENSG00000253948.1_5	5.4	7.2	4.2	3.3	divergent to promoter of *VPS13B*	26358774, 32605629
ENSG00000272686.1_4	1.7	1.9	1.5	1.5	divergent to promoter of *WASL*	
ENSG00000248092.7_5	4.8	4.9	3.4	4.2	divergent to promoter of *NNT*	16804088, 17922105
ENSG00000237989.1_3	0.3	0.5	0.5	0.7	divergent to *AP001046*.*1*	

The FPKM value and genomic information for each lncRNA are shown. The PubMed ID (PMID) of up to two of the most relevant references for those lncRNAs whose neighboring genes have been shown to be involved in the pathogenesis of diabetes have also been reported.

When the genomic context of these lncRNAs was analyzed, it was discovered that most were likely to act in some regulatory capacity in these cells (Fig 2B). Of these, *MZF1-AS1*, *DPP9-AS1*, and *PINK1-AS* were shown to share a portion of overlapping sequence with their neighboring antisense protein-coding genes, myeloid zinc finger 1 (*MZF1*), dipeptidyl peptidase 9 (*DPP9*), and PTEN induced kinase 1 (*PINK1*), respectively. Importantly, the expression of MZF1-AS1 and DPP9-AS1 showed a very high positive correlation with MZF1 and DPP9, respectively (S1 Fig). This suggests that these lncRNAs may form an RNA duplex via interactions between these antisense transcripts to stabilize the expression of the corresponding protein-coding gene. In addition, the promoter sequence of *MZF1-AS1* could also act as the promoter for the nearby gene, ubiquitin conjugating enzyme E2 M (*UBE2M*). This shared promoter sequence was also observed in *VPS13B-DT* and *NNT-AS1* which share a common promoter sequence with their neighboring protein-coding genes, vacuolar protein sorting 13 homolog B (*VPS13B*) and nicotinamide nucleotide transhydrogenase (*NNT*), respectively. Thus, these lncRNA-protein gene pairs may be affected by a common signaling pathway. Finally, *MIR137HG* contains the miR-137 sequence within its intronic region suggesting that MIR137HG lncRNA is likely the primary miRNA (pri-miRNA) for miR-137. Previous reports suggest that the suppression of miR-137 in endothelial cells ameliorates high glucose-induced oxidative stress by de-repressing protein kinase AMP-activated catalytic subunit alpha 1 [25]. Thus, MIR137HG may play a significant role in regulating the metabolic response of specific cells to increased glucose and thus diabetes via its production of miR-137.

**Fig 3:**
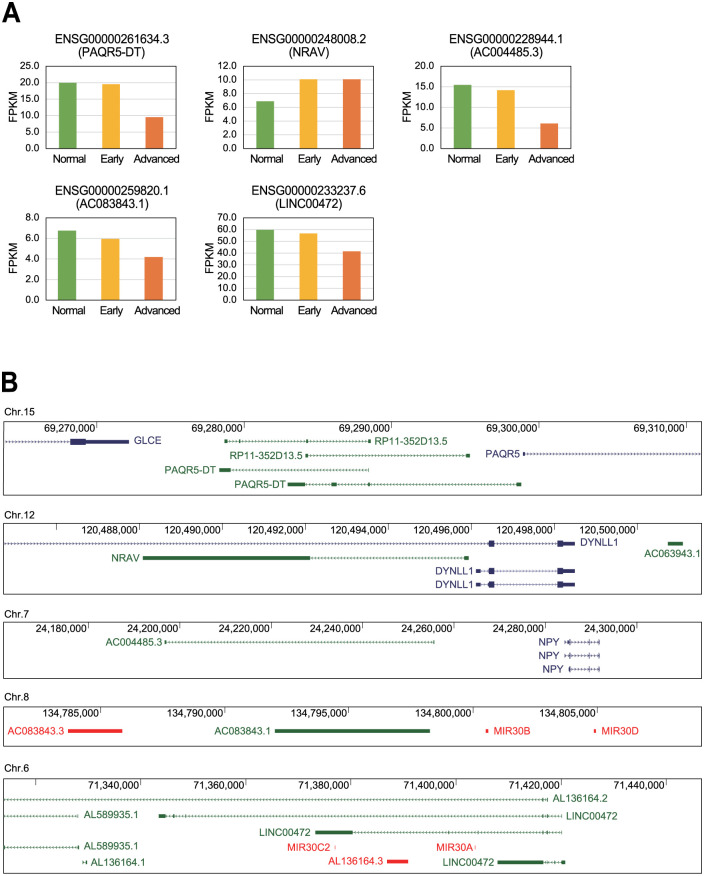
Selected dysregulated lncRNAs identified in the kidney tissues of patients with diabetes. (A) The differences in the expression of these lncRNAs in each group were calculated using the data from the public dataset GSE142025 and reported as FPKM values. (B) Schematics describing the genomic context of each of the lncRNAs as reported in Genome Browser. Blue lines represent protein-coding genes, green lines lncRNA genes, and red lines small non-coding RNA genes.

The kidney data was collected from the GSE142025 dataset which included biopsy samples from patients with diabetic nephropathy [23]. Evaluation of this data identified 15 significantly differentially expressed lncRNAs associated with advanced diabetic nephropathy (these included all the transcripts in the top 5% based on *P*-value and 20% based on average expression level) (Table 2). Of these, five had previously been described as related to diabetes (Fig 3) and of those only the negative regulator of antiviral response (NRAV) was shown to have increased expression in the sample from the patients with advanced diabetic nephropathy when compared to that in the control samples. The other four lncRNAs, including PAQR5 divergent transcript (PAQR5-DT), AC004485.3, AC083843.1, and long intergenic non-protein coding RNA 472 (LINC00472), decreased in the diabetic nephropathy samples (Fig 3A).

**Table 2:** Expression levels for each of the significantly altered lncRNAs in the kidneys of patients with diabetes.

Gene ID	Control	Early DN	Advanced DN	Description	Reference (PMID)
ENSG00000231312.6_5	11.8	10.7	6.9	divergent promoter to *MAP4K3*	
ENSG00000261634.3_4	20.0	19.6	9.5	*PAQR5-DT* (divergent promoter to *PAQR5)*	22933106
ENSG00000259291.2_4	47.7	51.7	25.6	antisense to intron of *ZNF710*	
ENSG00000231889.7_4	4.9	6.0	7.6		
ENSG00000248008.2_4	6.9	10.1	10.1	divergent promoter to *DYNLL1*	28321468
ENSG00000275234.1_4	16.1	18.3	11.3	antisense to intron of *DENND1C*	
ENSG00000228944.1_4	15.5	14.2	6.1	divergent promoter to *NPY*	7479313, 28423914
ENSG00000260658.5_5	20.8	18.4	11.0	intergenic	
ENSG00000226674.9_4	12.3	8.5	5.5	intergenic	
ENSG00000259820.1_4	6.8	6.0	4.2	miR-30b~30d host gene?	19096044, 30721562
ENSG00000267575.6_5	11.5	15.9	15.6	divergent promoter to *LINC00662*	
ENSG00000233237.6_3	59.8	56.7	41.5	miR-30a~30c-2 host gene	27221738, 30002134
ENSG00000242125.3_4	5.0	6.1	9.4	snoRNA host gene	
ENSG00000230551.4_5	7.3	5.3	5.6	overlap to protein-coding gene	
ENSG00000234456.7_3	34.5	29.4	20.4		

The FPKM value and genomic information for each lncRNA are shown. The PubMed ID (PMID) of up to two of the most relevant references for those lncRNAs whose neighboring genes have been shown to be involved in the pathogenesis of diabetes have also been reported.

The genomic context of these lncRNAs also provided some hints at their working mechanism (Fig 3B). This includes *PAQR5-DT*, *NRAV*, and *AC004485*.*3* which share a common promoter sequence with their neighboring genes, progestin and adipoQ receptor family member 5 (*PAQR5*), dynein light chain LC8-type 1 (*DYNLL1*), and neuropeptide Y (*NPY*), respectively. Furthermore, the lncRNA gene *AC083843*.*1* is close to *MIR30D* and *MIR30B* in the genome and has the same transcriptional direction suggesting that this lncRNA is a partial transcript of the pri-miR-30d~30b sequence. Finally, *LINC00472* includes both the *MIR30A* and *MIR30C2* genes within its sequence suggesting that this lncRNA may be the primary transcript for miR-30a and miR-30c. This is noteworthy as several reports have confirmed the importance of the miR-30 family of miRNAs in the pathogenesis of diabetes [26–29]. Therefore, lncRNAs AC083843.1 and LINC00472 could affect the progression of diabetes via their production and potential control of various miR-30 miRNAs.

**Fig 4:**
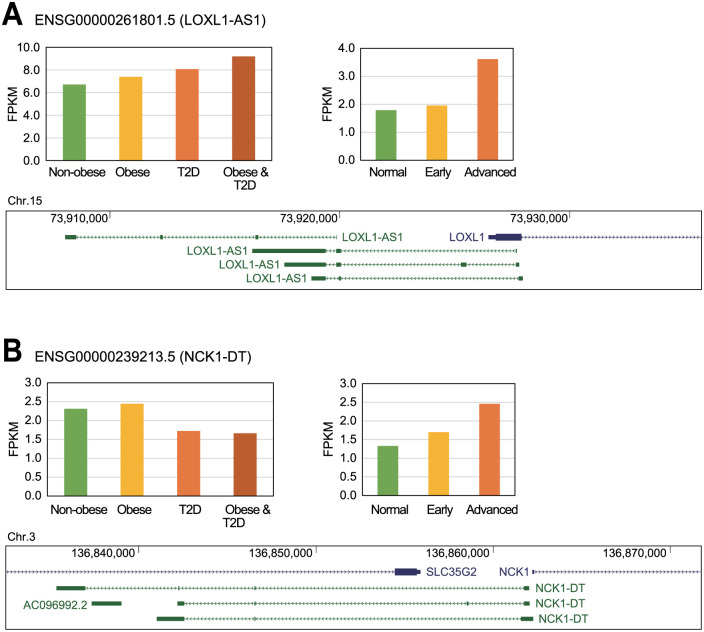
Commonly dysregulated lncRNAs identified in both myotube and kidney samples from patients with diabetes. Of the significantly altered lncRNAs from both the myotube and kidney tissues of the patients with diabetes, only (A) LOXL1-AS1 and (B) NCK1-DT were identified in both tissues and are shown here. The graphs describe the FPKM value for each lncRNA in each sample set and the genomic context for each is shown below. Blue lines represent protein-coding genes, green lines lncRNA genes, and red lines small non-coding RNA genes.

To identify any common lncRNAs within these datasets, those lncRNAs that were significantly dysregulated in both skeletal muscle and renal tissues of a patient with diabetes were selected. This analysis identified eight commonly dysregulated lncRNAs (Table 3), two of which, LOXL1 antisense RNA 1 (LOXL1-AS1) and NCK1 divergent transcript (NCK1-DT), had been previously associated with the pathogenesis of diabetes (Fig 4). LOXL1-AS1 lncRNA is increased in both myotube and kidney tissues in patients with diabetes (Fig 4A) and it has been reported that lysyl oxidase like 1 (*LOXL1*), the protein-coding gene neighboring LOXL1-AS1, has a common promoter sequence or overlaps with *LOXL1-AS1* as a sense-antisense pair depending on its isoform. However, there was no change in LOXL1 expression between control and diseased tissues from a rat model of diabetic nephropathy [30]. Thus, it is plausible that this lncRNA may use an alternative regulatory mechanism and may not influence diabetes via the direct regulation of its neighboring gene. NCK1-DT is an interesting lncRNA as it demonstrates opposing dysregulation patterns in myotube and kidney samples (Fig 4B). Thus, the expression of NCK1-DT decreased in the myotubes of diabetic patients when compared to the control, but its expression increased in the renal tissues of patients with diabetic nephropathy. This suggests that this lncRNA may work differently depending on the tissue or fine-tunes its target genes. A previous report suggests that NCK adaptor protein 1 (*Nck1*)-knockout pancreatic beta cells demonstrated improved survival in response to diabetes-related stresses [31], while another study from the same group suggested that the sequestration of Nck1 protein in pancreatic beta cells protects against diabetogenic stresses [32]. This suggests that the identification of the role of NCK1-DT in the pathogenesis of diabetes and the elucidation of its regulatory mechanism concerning NCK1 may be of significant interest in the future.

**Table 3:** Expression levels for each of the commonly dysregulated lncRNAs identified in both myotube and kidney samples from patients with diabetes.

Gene ID	Non-obese	Obese	T2D	Obese & T2D	Control	Early DN	Advanced DN	Description	Reference (PMID)
ENSG00000234432.4_4	0.2	0.3	0.4	0.3	0.8	1.2	1.3	intergenic	
ENSG00000285338.1_1	2.1	2.0	1.7	1.5	3.3	3.9	5.4	part of protein-coding gene	
ENSG00000261801.5_4	6.7	7.4	8.1	9.2	1.8	2.0	3.6	divergent to *LOXL1*	29207131
ENSG00000226674.9_4	0.3	1.0	1.5	1.5	12.3	8.5	5.5	intergenic	
ENSG00000273000.5_5	2.9	2.4	3.2	3.0	53.4	57.1	35.1	antisense to *RGL4*	
ENSG00000239213.5_4	2.3	2.4	1.7	1.7	1.3	1.7	2.5	divergent promoter to *NCK1*	26434994, 29941454
ENSG00000269996.1	16.6	15.6	12.7	13.9	2.7	1.0	1.1	part of protein-coding gene	
ENSG00000253738.1_4	15.3	17.9	13.9	14.3	5.7	3.6	3.6	divergent promoter to *OTUD6B*	

The PubMed ID (PMID) of up to two of the most relevant references for those lncRNAs whose neighboring genes have been shown to be involved in the pathogenesis of diabetes have also been reported.

## Conclusions

Here, a diverse range of dysregulated lncRNAs was identified from both the skeletal muscle and kidney tissues of diabetic patients, respectively, and also the common lncRNAs in these two tissues were discovered. By controlling the expression of lncRNAs presented in this study [33], it can be applied to observing diabetes-related phenotypes in animal models. Alternatively, by measuring the expression of these lncRNAs in body fluids including the blood of patients with diabetes, their possibility of application as a biomarker may be studied. Given that there are no studies comparing the roles of various lncRNAs in the different tissues associated with diabetic pathogenesis, the lncRNA lists reported in this study may help identify potential targets for future studies designed to elucidate the roles of lncRNAs in diabetic pathogenesis and the development of novel therapeutic interventions.

## Supporting information

S1 Fig Expression correlation between lncRNAs and their corresponding antisense protein-coding genes. (PDF)

S1 Table The expression level of lncRNAs in the skeletal muscle dataset used in this study. (XLSX)

S2 Table The expression level of lncRNAs in the kidney dataset used in this study. (XLSX)
