# Supplementary figures and images for: Comparing the long non-coding RNA expression profiles of skeletal muscle and kidney tissues from patients with diabetes
Source: PLoS One. 2022 Sep 26;17(9):e0274794. doi: 10.1371/journal.pone.0274794 (PMC9512191; doi:10.1371/journal.pone.0274794)

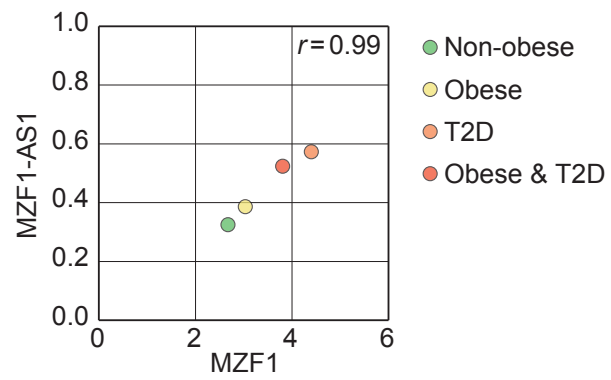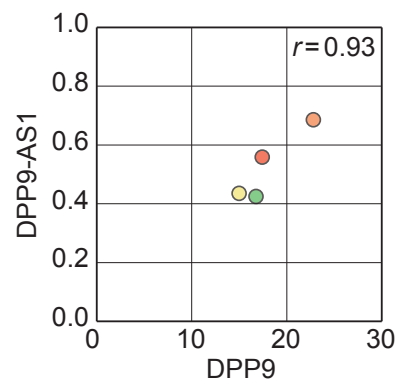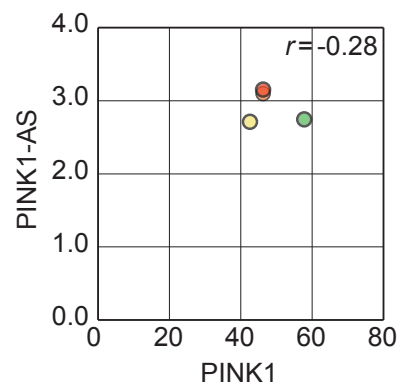

Supplement: S1 Fig — (PDF) [file pone.0274794.s001.pdf]
